# Autism paradigms in a developing country setting: Results and implications of a Zimbabwean study

**DOI:** 10.4102/ajod.v14i0.1638

**Published:** 2025-07-18

**Authors:** Jabulani Mpofu, Maximus M. Sefotho

**Affiliations:** 1 Department of Educational Psychology, Faculty of Education, University of Johannesburg, Johannesburg, South Africa

**Keywords:** autism spectrum disorders, paradigms, diversity, human variation, neurodiversity, pathology, young people, social justice

## Abstract

**Background:**

Autism spectrum disorders (ASD) are an evolving concept in the neurodiversity community. There is a continuum of views ranging from biological to social models, of which the medical model views ASD as pathological rather than atypical. How people live with their diversity attributes may depend on how they understand their own diversity attribute.

**Objectives:**

This study explored self-perceptions of young people with mild-to-moderate ASD within their cultural context.

**Method:**

Participants included an equal number of males and females with mild-to-moderate ASD (five each). They participated in two focus group discussions on self-perceptions of life situations in young people with ASD and whether they are considered as neurodiversity or pathology paradigms. Data analysis was done using the thematic content analysis approach.

**Results:**

Participants viewed ASD as: (1) a human neurological variation, (2) were not disordered, (3) had sense of friendship and belonging, and (4) had natural and normal social emotional development.

**Conclusion:**

Young people with ASD perceive ASD from a neurodiversity approach. A neurodiversity approach to ASD is primarily a social justice movement aiming to end what proponents see as the default pathologisation of neurodivergence and promoting the acceptance and accommodation of human neurodiversity.

**Contribution:**

This study enhances understanding of self-perceptions among young people with ASD in Zimbabwe, revealing challenges and strengths unique to their experiences. It may inform educators and policymakers, promoting inclusive practices and tailored interventions, ultimately fostering empowerment, acceptance, and improved quality of life for young people within this community.

## Introduction

People’s perceptions of diversity attributes may depend on various frames of reference. This is true for people with neurodevelopmental disabilities, such as autism spectrum disorders (ASD). Traditionally, studies on abnormal development have been carried out inside the confines of a medical model that sees disability as a condition that can, and more importantly, ought to be treated so that the person can get better and resume a ‘normal life’ (WHO 2016). Put differently, the medical paradigm attends to an individual with a neurodevelopmental disorder as someone who has a disease (e.g. measles). The medical model sees people with disabilities like ASD as helpless victims and dependents who need expert assistance and monitoring. Due to this, people with ASD have attempted to ‘camouflage’ their condition by making themselves seem more neurotypical (Mandy 2019). This has led to feelings of burnout, weariness, anxiety, sadness, stress, decreased well-being and suicidality (Cage & Troxell-Whitman 2019; Livingston, Shah & Happé 2019). The medical model perspective of disability seems to raise important concerns about its primary objectives, and this has led to the development of more communally accepted views of disabilities from inclusion movement groups. Inclusion movement groups are groups that aim to secure equity and rights for people with disabilities.

The medical model of disability was questioned by inclusion movements, who suggested replacing it with a neurodiversity approach to see ASD from a neurodiversity perspective as an alternative to the medical model. The neurodiversity approach to ASD is more related to an ‘ecological society’ where minority minds are valued according to their own niche (Cage & Troxell-Whitman 2019). The neurodiversity perspective model integrates elements of the social and medical models that are beneficial and blends diagnostic data with psychosocial elements of life, such as resilience. Rather than discounting the medical or social perspectives of disability, the neurodiversity perspective combines them with modern ideas of disability, health and function and is consistent with inclusive community practices (Mpofu 2024). The medical model is nevertheless essential to expanding knowledge of ASD, notwithstanding these critiques. New diagnostic instruments and biomarkers are being investigated as part of ongoing research in an effort to identify ASD more precisely (Cage & Troxell-Whitman 2019). Studies on single nucleotide polymorphisms and copy number variations are among other genetic advancements that are helping to clarify ASD’s hereditary components and providing guidance for possible future treatments (Livingston et al. 2019). Moreover, a thorough understanding of broad-spectrum illnesses associated with ASD and related disorders increasingly depends on interdisciplinary approaches that integrate knowledge from genetics, neurology, psychology and social sciences.

According to the American Psychiatric Association (APA 2013), ASD are chronic neurodevelopmental disorders marked by communication and social interaction deficits and limited behaviours, interests and activities beginning within the first 3 years of life. Autism spectrum disorders are typically diagnosed in childhood, with many noticeable symptoms emerging between the ages of 2 years and 3 years (Kenny et al. 2016).

The social model of disability has completely changed how society views and understands disability. The social model stresses how societal constraints contribute to disablement, unlike the medical model that considers conditions like ASD to be a personal tragedy or individual handicap (Dunn & Andrews 2015) It holds that barriers related to social, environmental and attitude are what cause impairment rather than being a feature of the person alone (Happé & Frith 2020). To accommodate a range of talents and promote inclusion, the emphasis should move from repairing the individual to changing social institutions (Silberman & Neuro 2015). The social model requires a distinction between disability and impairment. The social model advances that disability arises from the obstacles faced by individuals in societies, impairment refers to any physical, sensory, intellectual or psychological differences that an individual may have (Silberman & Neuro 2015) and emphasises that impediments to communication, mobility and attitude all contribute to the construction of disability within society (Happé & Frith 2020). The social model of disability emphasises that rather than being a product of personal failings, ASD results from society’s inability to accept variety (Happé & Frith 2020). This perspective shifts the focus from ‘fixing’ or ‘curing’ people with ASD to society’s need to provide accessible settings and opportunities. A culture of acceptance and respect is fostered by encouraging people to see those with ASD as an identity that is as legitimate as any other identity (Happé & Frith 2020). Through acknowledging and appreciating the abilities and qualities of people with ASD, society may progress toward a future that is fairer and more inclusive, empowering young people to develop a positive self-perception.

### Autism spectrum disorder paradigms in African contexts

The ASD paradigm provides a cogent ‘story’ of a meaningful, functional, yet culturally subjective reality (Ugwu, Ekere & Ohoh 2021). Autism spectrum disorders paradigms are discrete and culture-specific (Mohajan 2018; Ntuli 2018) and are affected by an individual’s background information, like occupation and orientation towards culture (Ugwu et al. 2021). The ASD paradigm as recognised in Western nations is not unique to any African country, nor do any African languages have words similar in meaning. However, most African cultures share the same paradigm of ASD with Western culture in that they perceive it as an illness, but they differ in how it is treated (Bakare & Munir 2011).

Autism spectrum disorders are known as *Usonji* in Kiswahili (Bakare & Munir 2011), meaning ‘disorders’. The causes of these disorders are witchcraft-related, but people from Kiswahili-speaking cultures believe it is curable by just having more and more interaction of the diagnosed with many people via frequent conversations. The Kiswahili perception of ASD borrows a lot from the social model of disability. It emphasises social functioning rather than pathological deficits (Bakare & Munir 2011). In Malawi, the Chewa people refer to people with ASD as *Osatha kukamba bwino*, meaning ‘someone who can’t speak properly’ (McFall 2016). The cause of ASD in Chewa culture is genetic or witchcraft. In isiZulu, ASD are known as *ukungashintshiswa* (McFall 2016). Its literal English translation is ‘not being changed’ implying a condition or state that cannot be altered or modified. Like in most African cultures, the Zulus believe that ASD are attributed to various factors such as ancestral spirits or curses. In Zulu culture, the treatment approaches for ASD include traditional healing, such as spiritual rituals to address the perceived underlying spiritual causes. The Maasai of Kenya do not have a term for ASD but the closest Maasai word *almodai* corresponds to the Western understanding of ASD, which means ‘stupid’ (Mpofu 2020). Also, in Zimbabwe, there are no terms in Shona, Ndebele or Kalanga that correspond to the Western definition of ASD (Mpofu 2020). The term ‘haana kukwana’ in Shona that most closely resembles ASD is ‘not enough’. A person who is inadequate could find it difficult to participate in social activities and could require assistance.

Autism spectrum disorders have a substantial impact on a young person’s development, leading to a range of social, emotional and cognitive difficulties, often with unique social deficits that affect their capacity to build relationships with their peers and disruptions in their emotional development (Chapman 2019, 2020). Among many issues, young people with ASD tend to have less developed social skills than their neurotypical peers, which might exacerbate feelings of loneliness and anxiety (Kasari et al. 2012) and may also have cognitive abnormalities, which are frequently typified by uneven cognitive profiles (Brown 2014; Ozonoff et al. 2015). Some of these developmental implications can be lessened by early intervention, specialised teaching methods and social skills training (Kasari et al. 2012). Therefore, creating successful intervention programmes that can promote the development and well-being of young people with ASD requires an understanding of the complex developmental effects of ASD (Lubin 2015).

Our study investigated self-perceptions of Zimbabwean young people with mild to moderate ASD in the context of their culture.

## Research methods and design

### Study design

This qualitative study (Kekeya 2019; Kivunja & Kuyini 2017) facilitated the exploration of self-perceptions of Zimbabwean young people with ASD in their cultural context using a variety of data sources (Creswell 2014; Dewi 2021; Kekeya 2019).

### Participants and setting

Participants included an equal number of males and females with mild-to-moderate ASD (five each). Table 1 provides additional demographic details. Pseudonyms were used to protect identity and ensure confidentiality.

**TABLE 1:** Demographic information of research participants.

Serial number	Gender	Names	Age (years)	ASD level and age at diagnosis	School or working	Family structure
1	Male	Takudzwa	17	Diagnosed mild at 4 years	School	Both parents
2	Female	Shamiso	14	Diagnosed moderate at 6 years	School	Single parent
3	Female	Lister	15	Diagnosed mild at 7 years	School	Both parents
4	Female	Taurai	19	Diagnosed mild at 4 years	Not working	Both parents
5	Male	Munetsi	21	Diagnosed mild at 8 years	Not working	Single parent
6	Female	Pride	18	Diagnosed mild at 8 years	School	Both parents
7	Female	Nyasha	20	Diagnosed mild at 5 years	Not working	Both parents
8	Female	Marble	17	Diagnosed moderate at 7 years	School	Both parents
9	Male	Taku	19	Diagnosed mild at 10 years	School	Both parents
10	Male	Tatenda	18	Diagnosed moderate at 9 years	School	Single parent

ASD, Autism spectrum disorders.

### Sampling procedure

Snowball sampling was used to recruit participants (Kekeya 2019). As participants referred others within the ASD community, we effectively expanded our sample size while maintaining relevance and diversity in experiences, resulting in rich qualitative data that accurately reflects the self-perceptions of youths with ASD (Creswell 2014).

### Data collection

Participants responded to one question based on how young people with ASD constructed their views and experiences of their self-perceptions. Two focus group discussions consisted of five participants each. The focus group discussant (first author) recorded the discussions. To increase the credibility of our study, we extended participant involvement by including them in focus group discussions that lasted for 2 h (Alharahsheh & Pius 2020; Roestenburg, Strydom & Fouche 2021). To get a fuller view of the phenomenon we were studying, we also triangulated the two focus groups’ information (Roestenburg et al. 2021). The verbatim recordings were read to the participants to ensure that they were accurately recorded and that the participants were satisfied with the recordings (Roestenburg et al. 2021).

### Data analysis strategies

Thematic content analysis was employed to systematically analyse the data (Kleinheksel, Rockich-Winston, Tawfik & Wyatt 2020). Initially, the transcripts from the two focus groups were coded using an inductive approach, identifying salient themes and patterns related to self-perception (Creswell 2014). Each segment of data was scrutinised for recurring concepts, leading to the development of initial codes. These codes were then organised into broader themes that encapsulated the lived experiences of the participants (Creswell 2014). The rigorous application of the constant comparative method facilitated the refinement of these themes, ensuring they were representative of the diverse perspectives within the sample. This coding process highlighted the nuanced complexities of self-perception formation among young people with ASD.

### Ethical considerations

The study received ethical approval from the Zimbabwe Open University on 05 April 2023 (Ref no. 6009/23). Informed consent, from parents for participants below 18 years, was obtained and assent from these participants.

## Results

Through their story-telling, the participants expressed that ASD represents: (1) human variation, (2) it is not a disorder, (3) have a sense of friendship and belonging, and (4) normal and natural social emotional development. The themes are presented and supported by the participants’ verbatim accounts.

### Theme 1: Autism spectrum disorder is a human neurological variation

The participants perceived that they look similar to their peers without ASD. They explained that all people were different and they expressed that individual differences are normal human variations not something which can be classified as an adverse condition. They also reported that being different was normal. However, the participants expressed that ASD was a handicapping condition. Participants reflected that ASD is not a disability and people with ASD must be seen like any other person without a disability. The following example explains participants’ reflections on ASD as a human neurological variation. Some of the participants said:

‘I have ASD. It’s a condition I live with it. It gives me some challenges here and there. I was born with this condition. Every person has some form of conditions and people respond differently to environmental stimuli … My condition is just like other conditions including those without disabilities. Thus, how I was created by God.’ (Munetsi, male, 21 years, mild, not working, single parent)

‘I have ASD it’s in my body. I was created like this and people must accept me like this. People are different.’ (Taurai, female, 19 years, mild, not working, both parents)

‘I am a person first. I am not feeling any pain as a result of ASD and I am comfortable with it the way Susan, Peter or Sarah live without ASD. We are human and humans are different.’ (Lister, female, 15 years, mild, school, both parents)

‘ASD is a condition like any other different conditions in our communities John is different to Peter and so on that’s what’s in the world. I am not denying that it is not disabling but I want you to take note that people are different in this community.’ (Taku, male, 19 years, mild, school, both parents)

‘A I see it ASD is real. I was diagnosed of ASD when I was young. The condition has its short comings but not in all facets, I can do other things perfectly better than my siblings without ASD or any other youths in our community and need help in others.’ (Pride, female, 18 years, mild, school, both parents)

### Theme 2: Autism spectrum disorder is not a disordered condition

Participants revealed that young people with ASD are not a disordered population. They explained that ASD is a disabling condition not a natural disorder. One of the participants, Munetsi, said that he does not receive special treatment in his community because of having ASD meaning he has order and not disorders. They reflected that ASD is better defined as a unique way of seeing the world rather than a disorder. This position is then consistent with the neurodiversity movement, which maintains that neurological variances, including ASD, are natural components of the human experience rather than pathological deficits. Participants said they look like any other young people in their communities and go along well with them. The following are quotes supporting this theme:

‘I have order I am not a disorder. I only have ASD and it’s not a disorder. I am not given special treatment at hospital because of ASD but I’m treated like any other person not a person who is disordered.’ (Munetsi, male, 21 years, mild, not working, single parent)

A participant said the following, while laughing:

‘kkkkkkk [*laughing*] I think I am fine. I look like any other youth in our community. I’m not disabled. I’m just different to Susan my friend but we go along very well.’ (Takudzwa, male, 17 years, mild, school, both parents)

One also said:

‘I think ASD is like any other condition.’ (Tatenda, male, 18 years, moderate, school, single parent)

Another nodded and said:

‘I have nothing to add what they all said is true.’ (Marble, female, 17 years, moderate, school, both parents)

### Theme 3: Sense of friendship and belonging

Participants revealed that young people with ASD have a sense of friendship and belonging. They narrated that they have friends at home and school like any other youth in their communities. They also said that they belong to various organisations in their communities, and the decision to have only a few friends rests with them. However, they also said some members of their communities were not willing to befriend them because of having ASD. At a family level, they revealed that they are sources of joy. They also said they were learning a lot from their affiliate organisations, and their contribution to them makes them proud. These quotes support this theme:

‘My best friend is my little sister we play a lot together. My other family members are friends too but not as close as my sister. I go to school mix with friends and this gives me opportunities to learn from others.’ (Takudzwa, male, 17 years, mild, school, both parents)

‘I have few friends but they are enough. I don’t need a lot of them … I’m a committee member of ASD youth organisation. In this group I learn a lot and this is good to me and others.’ (Taurai, female, 19 years, mild, not working, both parents)

One participant contributed by saying:

‘My friends are few the reason is others don’t like me because I am having ASD.’ (Shamiso, female, 14 years, moderate, school, single parent)

Another echoed:

‘I am a source of joy in my family. I always act in a manner that attract attention from every member of our family. I’m loved. I love all of them too.’ (Nyasha, female, 20 years, mild, not working, both parents)

### Theme 4: Natural and normal social emotional development

The participants revealed that their social emotional development is natural and normal. They explained that like neurotypical persons, people with ASD are capable of feeling negative social emotions, but they may choose to communicate those feelings in various ways. However, they revealed that persons with ASD have normal social emotional conditions. They also explained that people have different emotional states and that is natural and normal. They elaborated that when people with ASD experience negative emotions or feelings, communities view them as abnormal. The following are extracts from their narratives:

‘Just like neurotypical persons, people with are capable of feeling emotions, but they may choose to communicate those feelings in various ways.’ (Taku, male, 19 years, mild, school, both parents)

‘My social emotional development is stable. I react to social issues like any other person in my community.’ (Tatenda, male, 18 years, moderate, school, single parent)

Another participant added:

‘ASD is natural and normal, but when people with ASD experience emotions or feelings, they struggle to change their feelings because they often feel them so strongly and it makes them viewed abnormal.’ (Nyasha, female, 20 years, mild, not working, both parents)

## Discussion

Young people with ASD perceived themselves as having a human neurological variation. They explained that people are different and individual differences are normal. These findings suggest that instead of considering ASD as a disease the condition must be seen as a neurological variant, highlighting its unique neurobiological foundation. Autism spectrum disorders being seen as one of many human variations rather than strictly as a deficit has significant implications for young people with ASD in Africa. According to Odom et al. (2021), reframing ASD as a variation allows for a more inclusive approach that emphasises the strengths and capabilities of individuals with ASD. This perspective fosters a more accepting community, promoting the idea that neurodiversity is a vital aspect of human experiences and identities (Odom et al. 2021). In an African context, where cultural perceptions of disability can sometimes lean towards stigma and exclusion, this shift in viewpoint can lead to improved social integration and support systems for young people with ASD. A study by Agyei and Owusu (2022) highlights that when young people with ASD are viewed as part of the diverse spectrum of human variation, they are more likely to experience supportive environments that acknowledge their unique strengths. In this paradigm, educational interventions that celebrate diversity rather than focusing solely on remediation become essential. Furthermore, embracing ASD as a variation can positively influence self-perception among young people, contributing to higher self-esteem and reduced internalised stigma. These shifting views facilitate a greater sense of belonging and community engagement for young people with ASD in Africa. Thus, there is a pressing need for advocacy that focuses on educating communities about ASD as a natural human variation, which in turn could lead to transformative change in policies and practices that affect the lives of young people with ASD in African contexts. Such efforts are likened to a ripple effect, generating wider societal acceptance and paving the way for better integration of neurodiverse individuals into everyday life, ultimately enhancing their quality of life and sense of agency. The discourse surrounding ASD in Africa is gradually evolving, and embracing the neurodiversity paradigm holds promise for significant positive outcomes for young people with ASD.

Young people with ASD do not see themselves as being a disordered population. Although ASD is a disabling condition, it is a natural condition and not a disorder. Autism spectrum disorders is better defined as a unique way of seeing the world rather than a disorder. This position is consistent with the neurodiversity movement, which maintains that neurological variances, including ASD, are natural components of the human experience rather than pathological deficits. Autism spectrum disorders is better defined as a unique way of seeing the world rather than a disorder. Autism spectrum disorders as a nondisorder poses significant implications for societal attitudes, personal identity and policy development. Studies indicate that individuals with ASD who perceive their condition as neurodiversity rather than a disorder report heightened self-esteem and better mental health outcomes (Milo et al. 2020). Tewolde et al. (2021) highlight that when young people with ASD are framed as part of a diverse cognitive spectrum, they feel empowered to embrace their identities, countering stigma and fostering a sense of community. This reframing challenge prevailing narratives that often depict ASD solely in terms of deficits and limitations. Furthermore, it encourages more supportive environments; young people with ASD who view ASD positively exhibit increased social engagement and improved adaptive skills, suggesting a correlation between self-perception and social outcomes (O’Grady et al. 2019). In many African contexts, cultural perceptions play a crucial role in how ASD is understood. For instance, in Nigeria, Brand et al. (2022) found that families who align their understanding of ASD with the neurodiversity paradigm often become advocates for inclusive education policies. This advocacy not only leads to better educational opportunities for individuals with ASD but also raises awareness about ASD in broader societal contexts, thus challenging misconceptions.

Young people with ASD have a normal sense of friendship and belonging. They belong to various organisations in their communities and learn a lot from their affiliate organisations and their contribution to them makes them proud. However, some members of their communities were not willing to befriend them because of having ASD, although ASD does not seem to be a barrier to friendship and belonging. Communities need to understand that young people with ASD should be allowed to take part in community activities and to develop genuine relationships with people without disabilities. Studies on ASD in Africa highlight the profound implications of viewing ASD as possessing normal senses of friendship and belonging. In many African societies, ASD has historically been stigmatised, leading to significant challenges for individuals and families (Siddiqi, Ahmed & Banerjee 2019). However, a growing body of literature suggests that reframing ASD as a social asset can foster acceptance and promote inclusivity. Olusanya et al. (2021) found that approximately 70% of participants with ASD expressed a desire for community engagement when provided with supportive environments. This desire underscores the importance of integrating social value into perceptions of ASD, advocating for the recognition of the unique skills and perspectives that young people with ASD can contribute to society (Davis & Smith 2020). When communities in Africa begin to embrace this perspective, it enhances the self-perception and social participation of young people with ASD and challenges ingrained stereotypes that detract from their abilities. Chibanda, Cowan and Moosa (2022) revealed that, in communities where ASD is viewed positively, young people report increased participation in social activities, leading to improved mental health outcomes and a stronger sense of belonging. The notion of social value encourages collaborative efforts between families, educational institutions and government agencies to create inclusive policies and programmes. For instance, initiatives like inclusive education models have gained traction, providing tailored support that empowers youths with ASD to thrive academically and socially (Mokoena & Lewis 2023).

Young people with ASD perceive their social emotional development as natural and normal. Like neurotypical persons, people with ASD can feel emotional disturbances, and likewise they communicate these feelings in various ways which must not be misconstrued as being abnormal. The findings suggest that the concept of ‘a normal’ does not exist and people’s brains work differently, and there are differences in neurological development and function among individuals. This suggests that people should acknowledge, tolerate and accept a range of social emotional development variations. Viewing social emotional development in young people with ASD as natural and normal rather than as disorders requiring intervention can have significant implications for young people with ASD in Africa (Rowley 2020). This perspective fosters acceptance and reduces stigma, which is particularly critical in cultures where disability is often misunderstood. Nwokolo et al. (2018) emphasise that reframing ASD as a natural variation in human neurodiversity allows for a more inclusive societal approach. When young people with ASD perceive their condition as a unique aspect of their identity, it enhances their self-esteem and psychological well-being (Nwokolo et al. 2018). In many African countries, there exists a gap in understanding ASD, often compounded by cultural beliefs that can demonise or romanticise disability (Abubakar et al. 2020). Promoting an understanding of ASD as a natural condition can empower young people with ASD to challenge prevalent stereotypes and advocate for their rights (Gabriel et al. 2022). Additionally, when young people view their ASD as part of natural human diversity, they are more likely to engage with peers and build social networks, which has been shown to mitigate feelings of isolation (Murray et al. 2021).

A greater change in focus of the professional and research community from observing the pathology of ASD to the previously discussed neurodiversity paradigms has enormous implications for ASD. Further exploration of the impact of neurodiversity paradigm on the lives of young people with ASD may lead to the development of broader social and community resources for people with ASD. At the same time, a deeper understanding of neurodiversity paradigm can change how existing services are currently provided to people with ASD. In addition, analysing how the neurodiversity paradigm affects the lives of persons with ASD may aid in the development of strategies to enhance public comprehension of ASD and dispel preconceived notions and historical stigma. Consequently, this may contribute to the well-being of young people with ASD.

### Limitations

A major limitation of this qualitative study was the small sample size and geographical restriction. With only two focus groups of five participants each, the findings may not be representative of the broader population of young people with ASD, limiting generalisability. Additionally, the focus on a single geographical location may overlook cultural and socioeconomic factors that influence self-perception in diverse contexts.

## Conclusion

Our results showed that these young people with ASD are highly influenced by a neurodiversity approach to ASD. They see ASD as a human difference, that is natural. These findings suggest that, in addition to other sociodemographic variables, self-identity of the individual is important when evaluating the living situation of people with disabilities.
